# Identification and Functional Characterization of Divergent 3’-Phosphate tRNA Ligase From *Entamoeba histolytica*

**DOI:** 10.3389/fcimb.2021.746261

**Published:** 2021-12-17

**Authors:** Ruofan Peng, Shigeo Yoshinari, Tetsuro Kawano-Sugaya, Ghulam Jeelani, Tomoyoshi Nozaki

**Affiliations:** Laboratory of Biomedical Chemistry, Department of International Health, Graduate School of Medicine, The University of Tokyo, Tokyo, Japan

**Keywords:** RtcB, tRNA splicing, tRNA maturation, RNA ligase, translation, protist, evolution, *Entamoeba histolytica*

## Abstract

HSPC117/RtcB, 3’-phosphate tRNA ligase, is a critical enzyme involved in tRNA splicing and maturation. HSPC117/RtcB is also involved in mRNA splicing of some protein-coding genes including *XBP-1*. *Entamoeba histolytica*, a protozoan parasite responsible for human amebiasis, possesses two RtcB proteins (EhRtcB1 and 2), but their biological functions remain unknown. Both RtcBs show kinship with mammalian/archaeal type, and all amino acid residues present in the active sites are highly conserved, as suggested by protein alignment and phylogenetic analyses. EhRtcB1 was demonstrated to be localized to the nucleus, while EhRtcB2 was in the cytosol. EhRtcB1, but not EhRtcB2, was required for optimal growth of *E. histolytica* trophozoites. Both EhRtcB1 (in cooperation with EhArchease) and EhRtcB2 showed RNA ligation activity *in vitro*. The predominant role of EhRtcB1 in tRNA^Ile^(UAU) processing *in vivo* was demonstrated in EhRtcB1- and 2-gene silenced strains. Taken together, we have demonstrated the conservation of tRNA splicing and functional diversification of RtcBs in this amoebozoan lineage.

## Introduction

Transfer RNAs (tRNAs) are crucial adapter molecules involved in protein translation by delivering specific amino acids to translational machinery, according to a 3-nucleotide anticodon that complements the corresponding codon in messenger RNA (Grosjean, 2009). In all three domains of life, nascent tRNAs require numerous modifications to finally exert their functions in protein translation (Koh and Sarin, 2018). One of the vital tRNA modifications is intron splicing, by which some tRNA gene transcripts containing an intron are processed and matured to be fully functional (Hartmann et al., 2009; Popow et al., 2012). The intron splicing process of tRNA can be divided into two steps (Peebles et al., 1979). First, tRNA endonuclease excises a nascent pre-tRNA at two exon-intron boundaries with the products of two tRNA exon halves: the 5’-exon containing a 2’, 3’-cyclic phosphate at the 3’-end and the 3’-exon containing a 5’-hydroxyl at the 5’-end (Popow et al., 2012). Then, two tRNA exon halves are rejoined by a ligase to synthesize a mature tRNA molecule. tRNA endonucleases, responsible for the first step of tRNAs maturation, are well characterized. Their origin and the oligomeric architecture are well conserved in eukaryotes and their archaea (Trotta et al., 1997; Li and Abelson, 2000; Paushkin et al., 2004; Tocchini-Valentini et al., 2005). However, while the substrate recognition of archaeal tRNA endonucleases relies on the bulge-helix-bulge motif on pre-tRNA, eukaryal tRNA endonucleases cleave pre-tRNA at the conserved site (Reyes and Abelson, 1988; Thompson and Daniels, 1988; Abelson et al., 1998). On the other hand, molecular mechanisms of the second step, tRNA ligation, are significantly diversified between three domains of life (Silber et al., 1972; Konarska et al., 1981; Filipowicz and Shatkin, 1983; Greer et al., 1983a; Laski et al., 1983; Kjems and Garrett, 1988).

Mechanisms of tRNA ligation were first characterized in yeast (Greer et al., 1983b; Phizicky et al., 1986) and plants (Englert and Beier, 2005). The ligases that connect a 5’-phosphate to a 3’-hydroxyl moiety are called 5’-phosphate ligases, while the ligases that can directly utilize the 2’, 3’-cyclic phosphate to attack a 5’-hydroxyl terminus and form a 3’, 5’-phosphodiester, are named 3’-phosphate ligases (Yoshihisa, 2014). Those two enzymes, 5’-phosphate and 3’-phosphate ligases, are involved in tRNA ligation. The 5’-phosphate ligases were found in bacteriophages, archaea, eubacteria, trypanosomatids, yeast, and plants, while the 3’-phosphate ligases were demonstrated in a broad range of eukaryotes, archaea, and eubacteria (Yoshihisa, 2014). The central catalytic component of human 3’-phosophate ligase, HSPC117, is known to form a 200-kDa complex with DDX1, CGI-99, FAM98B, ASW, and archease (Popow et al., 2011; Popow et al., 2014). Indeed, archease, a protein that is conserved in all three domains of life, was reported to accelerate RtcB ligase activity in *Pyrococcus horikoshii* (Desai et al., 2014) and *Homo sapiens* (Popow et al., 2014). HSPC117 orthologs are also found in Opistokonta (vertebrates, lancelets, insects) and algae, but not in fungi and angiosperm, which instead possess Trl1 orthologs (5’-phosphate ligases) (Popow et al., 2012; Yoshihisa, 2014). In contrast to other RNA ligases, HSPC117/RtcB proteins require GTP rather than ATP as a reaction component to exert their ligating functions (Tanaka and Shuman, 2011; Chakravarty et al., 2012; Popow et al., 2014). Interestingly, HSPC117/RtcB orthologs were also found in Eubacteria, which does not require protein-assisted splicing since their tRNA genes containing self-splicing introns. Encoding RtcB in Eubacteria is assumed to be engaged with repair of damaged tRNAs and rRNAs under stress conditions (Tanaka and Shuman, 2011; Manwar et al., 2020).

*Entamoeba histolytica* is a protozoan parasite that belongs to the eukaryotic supergroup Amoebozoa and causes human amebiasis. Since it lacks morphologically discernible organelles typical to model eukaryotes, it was often regarded as one of the early-branching eukaryotes. Although the current genome information assures the presence of organelles such as the endoplasmic reticulum, the Golgi apparatus, endosomes, and lysosomes, the organism lacks canonical mitochondria due to anaerobic lifestyle (Mi-Ichi et al., 2009; Mi-Ichi et al., 2015). Such drastic changes in the fundamental architecture of cellular functions indicate a great value of the organism as an atypical eukaryote. To explore uniqueness and peculiarities of tRNA biology of this organism, we investigated tRNA repertoire in the genome and identified an extensive repertoire of tRNA genes of approximately 2,670 (Kawano-Sugaya et al., 2020). It was previously demonstrated that tRNA genes are often clustered in the genome of other eukaryotes (Tawari et al., 2008). In *E. histolytica*, almost all tRNA genes were found in arrays and adjacent to short tandem repeats (STR) except for five tRNA isoacceptor types, which were found to be dispersed in small numbers throughout the genome (Clark et al., 2006; Tawari et al., 2008). In the present study, we classified a whole repertoire of tRNA genes in *E. histolytica*, including intron-containing tRNA genes encoding 42 tRNA^Tyr^(GUA) and 52 tRNA^Ile^(UAU). We then investigated if the pathway for tRNA splicing exists in *E. histolytica.* The *E. histolytica* genome encodes two isotypes of HSPC117/RtcB (EhRtcB1 and EhRtcB2), which are distributed to different cellular localization, the nucleus, and the cytoplasm, respectively. The ligase activity of EhRtcBs was demonstrated *in vitro* by radioisotopic labeled RNA ligation assay for both EhRtcB1 and EhRtcB2. The *in vivo* ligase activity in *E. histolytica* was also demonstrated by the reduction of mature tRNA^Ile^(UAU) caused by *EhRtcB1* knockdown. We further demonstrated that the knockdown strains for *EhRtcB1*, but not *EhRtcB2*, showed a severe growth defect, suggesting the vital role of EhRtcB1 in proliferation. Altogether, these data validated the enzymological and biological functions of EhRtcB1 and EhRtcB2.

## Materials and Methods

### tRNA Intron Prediction

The whole genomic sequence of *E. histolytica* clonal strain HM-1: IMSS cl6 was applied to tRNAscan-SE (Lowe and Eddy, 1997; Lowe and Chan, 2016) to predict all tRNA sequences. All predicted tRNA sequences were counted and grouped according to corresponding codons.

### Phylogenetic Analysis

BLASTp search was performed to identify HSPC117/RtcB orthologs from a broad range of taxa by using *Homo sapiens* HSPC117 as a query. Obtained sequences were aligned by MAFFT v7.475 (Katoh and Standley, 2013) and trimmed by TrimAl v1.4 (Capella-Gutierrez et al., 2009), and phylogenetic reconstruction was conducted by the maximum likelihood method in FastTree 2.1.10 with default parameters (Price et al., 2010) with HSPC117/RtcB orthologs got from BLASTp searches.

### Plasmid Construction

Total RNA of *E. histolytica* trophozoites was extracted, and mRNA was purified. According to the manufacturer’s protocol, cDNA was synthesized by SuperScript III First-Strand Synthesis System (Invitrogen, Waltham, MA, USA). The protein-coding region of *EhRtcB1* (EHI_169260) and *EhRtcB2* (EHI_184560) were amplified by PCR from *E. histolytica* cDNA using appropriate oligonucleotides summarized in **Supplementary Table 1**. To express EhRtcB1 and EhRtcB2 fused with the hemagglutinin (HA) tag at the amino terminus in *E. histolytica* trophozoites, PCR fragments amplified from cDNA using appropriate primers were digested with StuI and SalI, purified, and ligated to SmaI- and XhoI-digested pEhExHA (Nakada-Tsukui et al., 2009) in blunt-end and compatible cohesive ends manner to produce pEhEx-HA-EhRtcB1 and pEhEx-HA-EhRtcB2. To express EhRtcB1 and EhRtcB2 fused with green fluorescence protein (GFP) at the amino terminus in *E. histolytica* trophozoites, PCR fragments amplified from cDNA using appropriate primers were digested with StuI and SalI, purified, and ligated to SmaI- and XhoI-digested pEhExGFP (Somlata et al., 2017) in blunt-end and compatible cohesive ends manner to produce pEhEx-GFP-EhRtcB1 and pEhEx-GFP-EhRtcB2.

For antisense small RNA-mediated gene silencing in *E. histolytica*, the fragments corresponding to the full-length protein of EhRtcB1 and EhRtcB2 were PCR-amplified from cDNA by using appropriate primers, digested by StuI and SacI, and cloned into StuI- and SacI-digested psAP2-Gunma (Mi-Ichi et al., 2011) to produce psAP2-Gunma-Eh-RtcB1-Full and psAP2-Gunma-Eh-RtcB1-Full. Subsequently, plasmids for gene silencing, which contained only a 264 bp or 299 bp fragment corresponding to the amino-terminal portion of EhRtcB1 and EhRtcB2, respectively, were also constructed by PCR amplification using psAP-2-Gunma-Eh-RcB1-Full or psAP-2-Gunma-Eh-RcB2-Full and appropriate primers, and self-ligated to produce psAP-2-Gunma-Eh-RcB1-Short and psAP-2-Gunma-Eh-RcB2-Short.

To express bacterial recombinant proteins of EhRtcB1 and EhRtcB2 fused with the histidine tag at the amino terminus, the protein-coding region of EhRtcB1 and EhRtcB2 was PCR-amplified using pEhEx-HA-EhRtcB1 or pEhEx-HA-EhRtcB2 as a template and appropriate primers. The fragments were cloned into NdeI- and XhoI-double digest pET28 a (+) using Infusion cloning kit (Clontech) to produce pET28a-N-His-EhRtcB1 and pET28a-N-His-EhRtcB1. For recombinant Archease, the protein-coding region of *E. histolytica Archease* gene (EHI_028560) was amplified using cDNA as template and appropriate primers, digested with NdeI and BamHI, and ligated to NdeI- and BamHI-digested pET15b to produce pET15b-EhArchease. Plasmid for expressing *E. coli* RtcB was a gift from Takashi Yokogawa, Gifu University.

### Culture and Transfection of *E. histolytica* Trophozoites

Trophozoites of the *E. histolytica* strain HM-1:IMSS cl-6 (Diamond et al., 1978) and G3 (Bracha et al., 2006) were maintained axenically in Diamond’s BI-S-33 medium (Diamond et al., 1978) at 35.5°C. The plasmids for expression of HA-tagged and GFP-fused EhRtcBs, generated as above, were introduced into HM-1 trophozoites by lipofection as previously established (Nozaki et al., 1999). For HA-tagged or GFP-fused EhRtcBs expressing strains, 5 or 20 µg/ml of G418 (Gibco/Life Technologies, Waltham, MA, USA) was used to achieve appropriate expression levels, respectively. The plasmids for gene silencing were introduced into G3 strain, and transfectants were maintained with 10 µg/ml of G418.

### cDNA Preparation in EhRtcB1 and EhRtcB2 Gene Silencing Validation

cDNAs from EhRtcB1-gs and EhRtcB2-gs strains were synthesized using 2 μg of total RNA (DNase I treated) and SuperScript III First-Strand Synthesis System (Invitrogen, Waltham, MA, USA) according to the manufacturer’s protocol.

### cDNA Preparation for tRNA^Ile^(UAU), tRNA^Tyr^(GUA), and tRNA^His^(GUG)

cDNAs of tRNA^Ile^(UAU), tRNA^Tyr^(GUA), and tRNA^His^(GUG) were reverse transcribed using 2 μg of total RNA [DNase I (Invitrogen) treated] and ReverTra Ace qPCR RT kit (TOYOBO, Japan) by tRNA^Ile^(UAU) common reverse primer, tRNA^Tyr^(GUA) common reverse primer, and tRNA^His^(GUG) reverse primer, respectively, according to the manufacturer’s protocol.

### Traditional PCR and Electrophoresis

cDNAs of tRNA^Ile^(UAU), tRNA^Tyr^(GUA), and tRNA^His^(GUG) were used as template for traditional PCR by using Pfu-X DNA polymerase (Jena Bioscience, Germany). tRNA^Ile^(UAU) and tRNA^Tyr^(GUA) common forward and reverse primers were used to synthesize both unspliced and spliced form of tRNA^Ile^(UAU) and tRNA^Tyr^(GUA), respectively, followed with 30 amplification cycles. tRNA^Ile^(UAU) common forward primer and spliced form-specific reverse primer were used to target the spliced tRNA^Ile^(UAU) specifically followed with 25 amplification cycles. tRNA^His^(GUG) is synthesized by designed primer followed with 30 amplification cycles. All the PCR products were run on 10% polyacrylamide gel, stained by 0.5 µg/ml EtBr.

### Sequencing of tRNA^Ile^(UAU) Transcripts

PCR products targeting tRNA^Ile^(UAU) transcripts were amplified using cDNA from psAP-mock strain by a common forward primer containing BamHI site and a common reverse primer containing XhoI site. Amplified PCR products were digested by BamHI and XhoI and ligated to a derivative of pET151/D-TOPO vector in which BamHI and XhoI sites had been engineered. Ligated plasmids were introduced into *E. coli*, and three to four colonies each for precursor (unprocessed) and mature (processed) tRNA^Ile^(UAU) transcripts were selected and sequenced by Sanger sequencing.

### Quantitative Real-Time PCR

The mRNA levels of *EhRtcB1* and *EhRtcB2* were measured by qRT-PCR, with RNA polymerase II gene (EHI_056690) as an internal control. Respective primers were used to target a 193 bp segment of *EhRtcB1*, a 226 bp segment of EhRtcB2, and a 204 bp segment of RNApolII. The amounts of transcripts of spliced tRNA^Ile^(UAU) were also quantitated by qRT-PCR. tRNA^His^(GUG) was used as a reference, which does not contain an intron in the *E. histolytica* genome based on the prediction by tRNAscan-SE. Respective primers were used to target a 52 bp segment of mature tRNA^Ile^(UAU) and a 70 bp segment of tRNA^His^(GUG). Twenty-fold diluted cDNAs from each strain were used as a template to interact with Fast SYBR Green Master Mix (Applied Biosystem, Foster City, CA, USA) according to the manufacturer’s protocol. qRT-PCR was conducted using StepOne Plus Real-Time PCR system (Applied Biosystems, Foster City, CA, USA). The data were analyzed by DataAssist software (ThermoFisher).

### Preparation of Recombinant Proteins

Plasmids for expressing recombinant EhRtcB1, EhRtcB2, *E. coli* RtcB and EhArchease, constructed as described above, were introduced into *E. coli* BL21 (DE3) cells by heat shock at 42°C for 1 min. Transformed *E. coli* cells were grown in 100 ml of Luria Bertani medium in the presence of 30 μg/ml kanamycin at 37°C. The overnight culture was used to inoculate 500 ml of fresh medium, and the culture was further continued at 37°C with shaking at 200 rpm. When A600 reached 0.4, cultures were chilled by ice, 2% of ethanol and 1 mM of isopropyl β-d-thiogalactopyranoside was added, and cultivation was continued for another 16 h at 18°C. *E. coli* cells from the induced culture were harvested by centrifugation at 8,000 g for 10 min at 4°C. The cell pellet was washed with Tris-saline (10 mM Tris pH 7.6 and 150 mM NaCl), resuspended in 40 ml of the lysis buffer (50 mM Tris–HCl, pH 7.6, 250 mM NaCl, and 10% sucrose, w/v) with 100 μg/ml lysozyme, 0.5 mg/ml E64, 1× Complete Mini protease inhibitor cocktail (Roche, Mannheim, Germany), and 1 mM phenylmethyl sulfonyl fluoride, and incubated at room temperature for 20 min, French press, added 0.1% Triton X-100 (v/v), and centrifuged at 25,000 g for 30 min at 4°C. The supernatant was mixed with 1 ml of 50% Ni^2+^-NTA His-bind slurry, incubated for 1 h at 4°C with mild rotating. The resin in a column was washed three times with Wash Buffer (50 mM Tris-HCl, pH 7.6, 2M KCl). Bound proteins were eluted with Elution Buffer [50 mM Tris-HCl, pH 7.6, 150 mM NaCl, 10% glycerol (v/v)] containing 100–500 mM imidazole to obtain recombinant EhRtcB1, EhRtcB2, *E. coli* RtcB, and EhArchease. After the integrity and the purity of recombinants, proteins were confirmed with 10% SDS-PAGE analysis, followed by Coomassie Brilliant Blue staining, they were extensively dialyzed twice against the 300-fold volume of 50 mM Tris-HCl, 150 mM NaCl, pH 7.6 containing 10% glycerol (v/v) for 18 h at 4°C. The dialyzed proteins were stored at –80°C with 10% glycerol in small aliquots until further use. Protein concentrations were spectrophotometrically determined by the Bradford method using bovine serum albumin as a standard as previously described.

### Growth Kinetics

Approximately 3×10^4^ exponentially growing trophozoites of *E. histolytica* G3 strain transformed with psAP2-Gunma-Eh-RtcB1-Full, psAP2-Gunma-Eh-RcB1-Short, psAP2-Gunma-Eh-RtcB2-Full, psAP2-Gunma-Eh-RcB2-Short, and psAP2G (control) were inoculated into 6 ml of fresh BI-S-33 medium containing 10 μg/ml geneticin, and the parasites were counted every 24 h on a hemocytometer.

### Assays for Enzymatic Activity

A synthetic RNA (5’-GUCGGUUCGUGCGAUGGUUGUAGC-3’) with 5’- and 3’ hydroxyl termini was labeled with 5’-[^32^P]-pCp by using T4 RNA ligase 1. The labeled RNA was purified through 7M urea-PAGE. RNA self-ligation assay mixture (total 10 μl) consisted of 2 μl of 5x RtcB assay Buffer [100 mM Tris-HCl (pH 7.6), 25 mM MgCl2, 5 mM MnCl2, 10 mM ATP, 5 mM GTP, 10mM DTT], 1 μl of RNase-OUT, 1 μl of RtcB solution containing various amount of the protein, 1 μl of EhArchease solution containing various amount of the protein, and the labeled substrate RNA with 100,000 cpm radioactivity. The assay mixture was incubated for 1 h at 37°C, and the reaction was stopped by adding 10 μl of FDE [90% (v/v) formamide, 20 mM EDTA, 0.02% (w/v) bromophenol blue, and 0.02% (w/v) xylene cyanol]. Approximately 5 μl of the assay mixture was electrophoresed by 7 M urea-PAGE. The radioactivity in gel was visualized by Phosphor Imager (Cytiva).

## Results

### The Repertoire of tRNAs in the *E. histolytica* Genome

**Figure 1:**
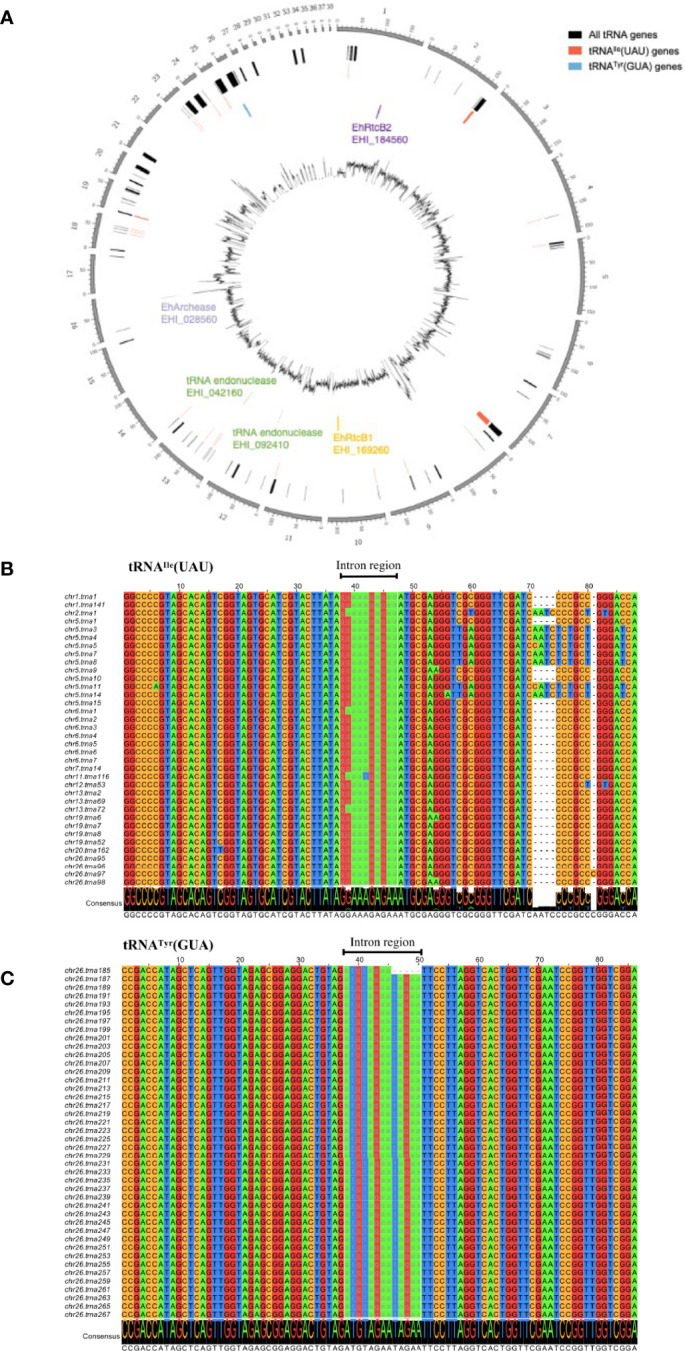
Graphical representation of the distribution of EhRtcBs, tRNA endonucleases, EhArchease, 52 tRNA^Ile^(UAU), and 42 tRNA^Tyr^(GUA) genes in the *E. histolytica* genome and alignment of the representative tRNA^Ile^(UAU) and tRNA^Tyr^(GUA) genes. **(A)** tRNA^Ile^(UAU) genes are scattered throughout the genome, while tRNA^Tyr^(GUA) genes are clustered on a single chromosome. Locations of genes encoding EhRtcB1, EhArchease, and two tRNA endonucleases are also shown. **(B)** Sixteen pseudo genes were removed from predicted tRNA^Ile^(UAU) genes. Thirty-six remaining tRNA^Ile^(UAU) were aligned by clustalW and drawn by Jalview. Chromosomal location and the assigned tRNA gene number are indicated. Nucleotides are color-coded. The intron regions are shown in lowercase and highlighted with gray font. **(C)** Forty-two predicted tRNA^Tyr^(GUA) were aligned as in **(B)**.

To see the repertoire of tRNA in the genome and also validate the significance of tRNA processing in *E. histolytica*, a comprehensive analysis of tRNA genes was conducted using our latest updated genome of HM-1 reference strain (Kawano-Sugaya et al., 2020) by tRNAscan-SE (Lowe and Eddy, 1997; Lowe and Chan, 2016) (**Table 1**). The genome of HM-1 apparently contains 2,670 tRNA genes. Among them, two groups of tRNA genes have a single intron: 52 tRNA^Ile^(UAU) and 42 tRNA^Tyr^(GUA) genes. These intron-containing genes represent approximately 3.8% of all tRNA genes. It is plausible that tRNA splicing is essential in *E. histolytica* because all tRNA^Ile^(UAU) and tRNA^Tyr^ (GUA) genes contain an intron. Note that an ortholog of tRNA endonuclease (two copied genes with 100% amino acid sequence identity to each other, EHI_092410, EHI_042160) and an ortholog of archease (EHI_028560) that has been known to be required for full enzyme activity of HSPC117 were also found in the *E. histolytica* genome. Chromosomal distribution of tRNA^Ile^(UAU), tRNA^Tyr^(GUA) genes are remarkably different (**Figure 1A**). While tRNA^Ile^(UAU) genes are scattered throughout the genome, tRNA^Tyr^(GUA) genes are clustered on a single chromosome. In addition, all 42 tRNA^Tyr^(GUA) genes are identical with one exception, whereas tRNA^Ile^(UAU) genes are quite polymorphic in both exon and intron regions (**Figures 1B, C**).

**Table 1:** Codon frequency and intron-containing tRNAs in *E. histolytica*.

Amino acid	Codon	Frequency	Anticodon	Total number	Intron
Phe	UUU	0.0%	AAA	1	0
	UUC	5.1%	GAA	137	0
Leu	UUA	3.9%	UAA	104	4
	UUG	3.8%	CAA	101	0
Ser	UCU	1.3%	AGA	35	0
	UCC	0.0%	GGA	0	0
	UCA	6.1%	UGA	163	0
	UCG	1.8%	CGA	49	0
Tyr	UAU	0.0%	AUA	0	0
	UAC	1.6%	GUA	42	42
Stop	UAA	0.0%	UUA	1	0
	UAG	0.0%	CUA	0	0
Cys	UGU	0.0%	ACA	0	0
	UGC	1.3%	GCA	35	0
Stop	UGA	0.0%	UCA	1	0
Trp	UGG	1.3%	CCA	35	0
Leu	CUU	0.7%	AAG	19	0
	CUC	0.0%	GAG	1	0
	CUA	0.5%	UAG	14	1
	CUG	1.9%	CAG	50	0
Pro	CCU	0.3%	AGG	8	0
	CCC	0.0%	GGG	0	0
	CCA	3.9%	UGG	103	0
	CCG	0.3%	CGG	8	0
His	CAU	0.0%	AUG	1	1
	CAC	1.5%	GUG	40	0
Gln	CAA	1.2%	UUG	31	0
	CAG	1.0%	CUG	27	0
Arg	CGU	6.3%	ACG	167	0
	CGC	0.0%	GCG	0	0
	CGA	3.4%	UCG	90	0
	CGG	0.0%	CCG	1	0
Ile	AUU	1.5%	AAU	39	0
	AUC	0.0%	GAU	0	0
	AUA	1.9%	UAU	52	52
Met	AUG	3.8%	CAU	101	0
Thr	ACU	1.9%	AGU	51	0
	ACC	0.0%	GGU	0	0
	ACA	3.9%	UGU	103	0
	ACG	1.2%	CGU	32	0
Asn	AAU	0.0%	AUU	0	0
	AAC	3.4%	GUU	90	0
Lys	AAA	1.4%	UUU	37	0
	AAG	3.3%	CUU	89	0
Ser	AGU	0.0%	ACU	0	0
	AGC	0.7%	GCU	19	0
Arg	AGA	1.6%	UCU	43	0
	AGG	1.2%	CCU	31	0
Val	GUU	0.0%	AAC	0	0
	GUC	5.1%	GAC	136	0
	GUA	0.6%	UAC	16	0
	GUG	0.5%	CAC	13	0
Ala	GCU	3.2%	AGC	86	0
	GCC	0.0%	GGC	0	0
	GCA	0.7%	UGC	20	0
	GCG	3.8%	CGC	101	0
Asp	GAU	0.0%	AUC	0	0
	GAC	6.9%	GUC	183	0
Glu	GAA	1.6%	UUC	42	0
	GAG	0.5%	CUC	14	0
Gly	GGU	0.0%	ACC	0	0
	GGC	1.6%	GCC	43	0
	GGA	2.2%	UCC	59	0
	GGG	0.2%	CCC	6	0
**Total**				2,670	100

### Phylogenetic and *In Silico* Analysis of HSPC117/RtcB Proteins

To further understand the conservation of tRNA splicing in *E. histolytica*, we investigated conservation of two core processes of tRNA splicing: cleavage and ligation of nascent tRNA transcripts. We first searched for *E. histolytica* HSPC117/RtcBs orthologs by BLASTp search using human HSPC117 as a query. Two HSPC117/RtcB orthologs, EhRtcB1 (XP_651200.1/EHI_169260) and EhRtcB2 (XP_648794.1/EHI_184560), were found in the *E. histolytica* HM-1 reference strain. EhRtcB1 and EhRtcB2 show 67.8% mutual amino acid identity, and 48.7 and 50.0% amino acid identity to *H. sapiens* HSPC117, respectively. To investigate the origin of *E. histolytica* RtcBs, 128 HSPC117/RtcB orthologs were aligned, and phylogenetic reconstruction was conducted (**Figure S1**). Only representative taxa from the eukaryotic supergroups were selected. Orthologs from animals other than *H. sapiens* were excluded because of high similarity and repetitiveness. RtcB orthologs from four *Entamoeba* species form two independent well-separated clades with a good bootstrap proportion, suggesting that two paralogs are conserved in four *Entamoeba* species. Furthermore, two *Entamoeba* clades, which form a monophyletic clade, are well separated from other eukaryotes, but all eukaryotes show monophyly independent orthologs of bacterial and archaeal origins. These data are consistent with the notion that *Entamoeba* RtcB is of eukaryotic origin and that the ancestral organism of *Entamoeba* possesses two isotypes of RtcB prior to the diversification of *Entamoeba* species, suggesting non-overlapping roles of both EhRtcBs. Similarly, *Trichomonas vaginalis* appears to possess four different RtcB orthologous genes, although gene multiplication is common in this organism (Alsmark et al., 2009; Aurrecoechea et al., 2009). Also, note that an ortholog of tRNA endonuclease was also found in the *E. histolytica* genome (two allelic genes encoding a protein of 100% amino acid identity to each other, EHI_092410, EHI_042160), but investigation on *Entamoeba* tRNA endonuclease is above the scope of this study.

Protein alignment of representative RtcBs (*E. histolytica, Pyrococcus horikoshii*, and *H. sapiens*) (**Figure S2**) shows a high degree of conservation of key residues implicated in the manganese binding and the guanylylation active site for *P. horikoshii* RtcB, which also supports the premise that EhRtcBs resemble mammalian/archaeal tRNA ligases and both EhRtcBs are functional. *In silico* survey of potential organelle targeting or localization motifs such as the signal peptide, the nuclear export signal, mitochondria targeting transit peptides, membrane targeting motifs such as the transmembrane region, and GPI anchor conjugation failed to detect significant motifs or domains. NLS was predicted to exist in EhRtcB1, but not EhRtcB2, by NLS Mapper (Kosugi et al., 2009) with a score of 4.5, indicating possible nuclear localization of EhRtcB1.

### Demonstration of tRNA Splicing in *E. histolytica* Trophozoites

**Figure 2:**
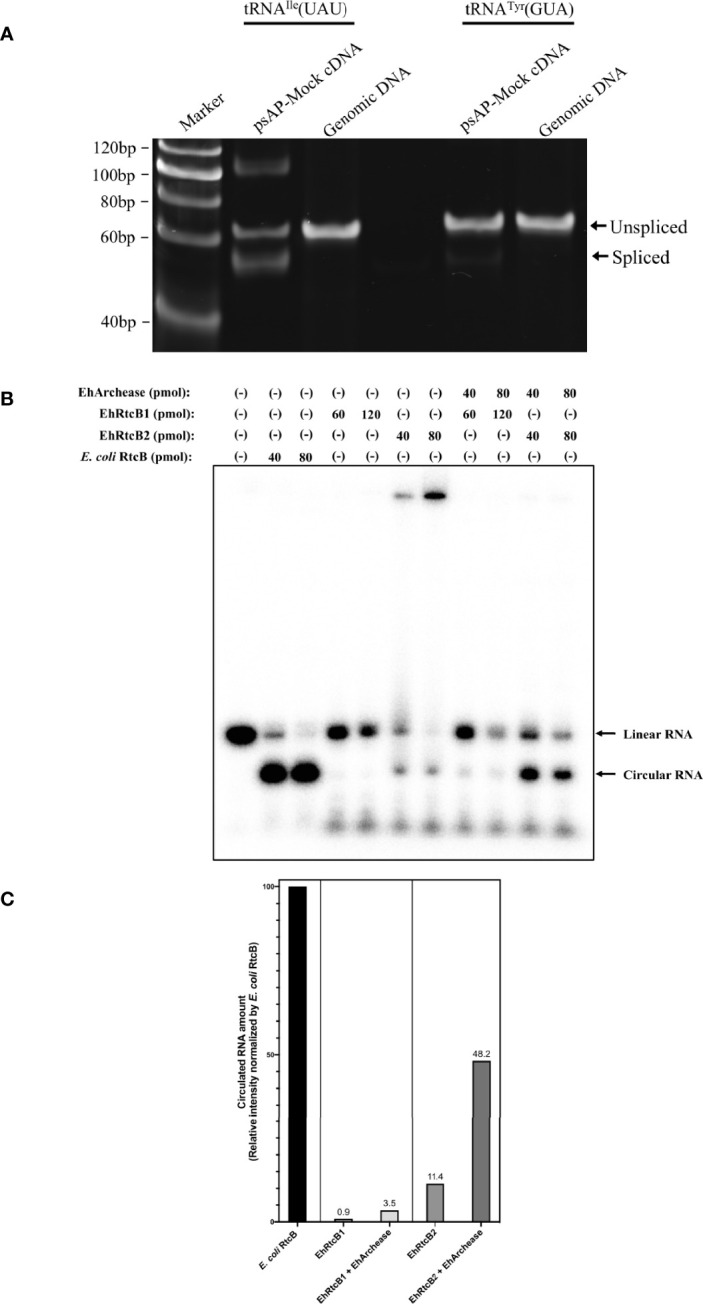
Verification of *in vivo* and *in vitro* EhRtcBs activity. **(A)** Verification of *in vivo* tRNA splicing of tRNA^Ile^(UAU) and tRNA^Tyr^(GUA) by RT-PCR. The tRNA splicing was examined by RT-PCR with common forward and reverse primers and polyacrylamide gel electrophoresis, by which both unspliced and spliced cDNAs from tRNA transcripts and genomic fragments were detected. RT-PCR with RNA samples without reverse transcriptase reaction failed to amplify corresponding bands in the same conditions (see **Figure 3B**). **(B)** Verification of *in vitro* RNA ligase activity by recombinant EhRtcBs. The assay mixture contained EhRtcB1, EhRtcB2, or *E. coli* RtcB in the presence or absence of EhArchease. RNA ligase activity of EhRtcBs and *E*. *coli* RtcB was monitored using ^32^P-labeled RNA substrate by RNA self-ligation assay. Linear and circular RNA are indicated. **(C)** Quantitation of circularized RNA. The intensity of the circularized RNA was measured by densitometric scanning of the gel image. The intensities of the bands corresponding to circularized RNA produced by EhRtcB1, EhRtcB2 only, EhRtcB1 plus EhArchease, or EhRtcB2 plus EhArchease are shown relative to that produced by *E*. *coli* RtcB. The normalized levels of circularized RNA are shown on each bar. Note that the ligase activity of EhRtcB1/2 was increased by EhArchease: relative EhRtcB1 and EhRtcB2 ligase activity (normalized against *E*. *coli* RtcB) were 11.4 and 48.2%, respectively, which were increased by 2.89 and 12.8-fold, respectively, compared to that without EhArchease.

To examine if both intron-containing tRNA, tRNA^Ile^(UAU) and tRNA^Tyr^(GUA), are spliced *in vivo*, the transcripts of the genes were amplified by PCR from cDNA from *E. histolyitca* control strain (psAP-mock transfected G3 strain), and their sizes were compared against those of the fragments amplified from the corresponding intron-containing genes, using genomic DNA as the template (**Figure 2A**). Common forward and reverse primers were designed to amplify both intron-containing and intron-spliced forms of tRNA^Ile^(UAU) and tRNA^Tyr^(GUA). One band corresponding to the intron-containing region of tRNA^Ile^(UAU) was amplified from genomic DNA, while three bands corresponding to intron-retained transcript (62 bp), intron-spliced transcript (52 bp), and the top band (~120 bp) of unknown sequence were amplified from cDNA. These PCR products from cDNA were cloned to a pET151/D-TOPO vector and sequenced as described in *Materials and Methods*. Sequencing results of precursor and mature tRNA^Ile^(UAU) are identical to the tRNA^Ile^(UAU) sequence predicted by tRNAscan-SE (**Figure S3**). Note that the ~120 bp band that had been amplified from cDNA and visualized in polyacrylamide gel electrophoresis was not visible in 3% agarose gel. Considering the partially self-complementary nature of tRNA, together with platform-dependent (agarose *vs* polyacrylamide) presence or absence of the ~120 bp band, we speculate that the ~120 bp band may be a differently annealed form or artifactual dimer of the main PCR product, probably easily denatured in the agarose gel electrophoresis, in which electrophoresis was carried out at higher temperature than polyacrylamide gel electrophoresis (**Figure S4**). Similarly, PCR amplified one band corresponding to intron-containing tRNA^Tyr^(GUA) fragment (65 bp) from genomic DNA, while two bands corresponding to intron-retained (65 bp) and intron-spliced (52 bp, faint) fragments were amplified from cDNA. However, the abundance of spliced tRNA^Tyr^(GUA) was lower than that of unspliced form, suggesting that most tRNA^Tyr^(GUA) remained unspliced *in vivo*.

### Demonstration of RNA Ligase Activity of Both EhRtcBs

To verify if both EhRtcB candidates possess RNA ligase activity, RNA self-ligation assay was performed (**Figure 2B**). Synthetic RNA possessing 5’- and 3’-hydroxyl termini were labeled with 5’-[^32^P]-pCp (**Figure S5**). EhRtcBs are expected to produce circular RNA, which migrates faster than linear RNA on urea-PAGE. As human tRNA ligase complex was previously reported to be stimulated by Archease, recombinant protein of *E. histolytica* Archease, whose encoding gene was found in the *E. histolytica* genome, was produced in *E. coli* and used for the RNA self-ligation assay. Circularizing activity by recombinant EhRtcB1 and EhRtcB2 was observed, although activity was weaker than that of *E. coli* RtcB, which was used as a positive control. The intensity of the circularized RNA was measured by densitometric scanning of the gel image (**Figure 2C**). The intensity of the bands corresponding to circularized RNA produced by EhRtcB1, EhRtcB2 only, EhRtcB1 plus EhArchease, or EhRtcB2 plus EhArchease is shown relative to that produced by *E. coli* RtcB. The ligase activity of EhRtcB1/2 was enhanced by EhArchease: relative EhRtcB1 and EhRtcB2 ligase activity (normalized against *E. coli* RtcB) were 11.4 and 48.2%, respectively, which increased by 2.89 and 12.8-folds, respectively, compared to that without EhArchease. The drastic increase in circular RNA produced by EhRtcB2 plus EhArchease was much higher than the increase found in a reaction with EhRtcB1 and EhArchease. Furthermore, archease was previously proven to interact with RtcB ligase and enhance its enzymatic activity. Archease itself failed to show RNA ligation ability, as previously shown (Desai et al., 2014). These data reinforce that both EhRtcBs possess RNA ligase activity (EhRtcB2>EhRtcB1), and their activity was enhanced by EhArchease, like human HSPC117. The slow migrating bands were visible only when the substrate was mixed with EhRtcB2, but not when mixed with both EhRtcB1/2 and EhArchease. We presume that the slow migrating band likely represents EhRtcB2/RNA complex. EhArchease presumably helped dissociation of the protein from the substrate.

### *In Vivo* Role of EhRtcBs in tRNA Processing

To further examine whether both EhRtcBs function as tRNA ligases *in vivo*, quantitation of nascent unprocessed, cleaved, and ligated forms of tRNA^Ile^(UAU) was performed by RT- and qRT-PCR using common forward primer [for non-spliced and spliced forms of tRNA^Ile^(UAU))] and either common reverse [for non-spliced and spliced forms of tRNA^Ile^(UAU))] or spliced form-specific reverse primer [for only the spliced form of tRNA^Ile^(UAU)] (**Figure S6**). Two primer sets were designed to specifically amplify only the spliced (cleaved and ligated) form or amplify both the spliced and unprocessed forms of tRNA^Ile^(UAU).

**Figure 3:**
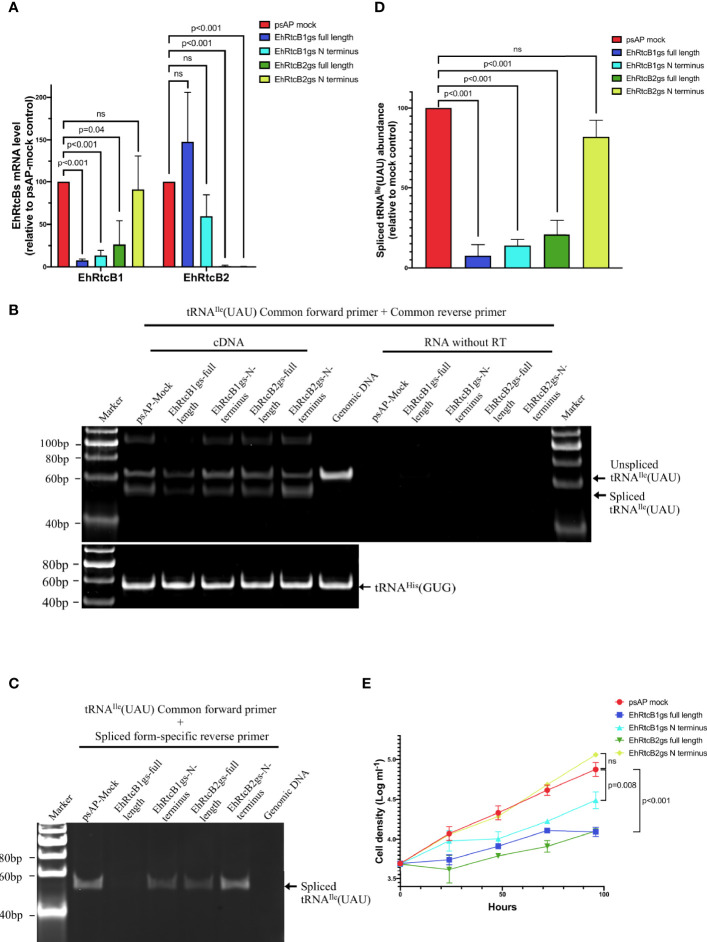
Effects of gene silencing of *EhRtcB*s on tRNA splicing and cellular proliferation. **(A)** Verification of repression of mRNA levels of *EhRtcB1* and *EhRtcB2* by gene silencing. Two *E*. *histolytica* strains each for *EhRtcB1* and *EhRtcB2* gene silencing were produced using gene silencing plasmid constructs containing either full-length or N-terminal portion of the protein-coding region of *EhRtcB1* and *EhRtcB2* genes, by small RNA-mediated transcriptional gene silencing, as described in *Materials and Methods*. The levels of mRNAs of each *EhRtcB1* **(A)** and *EhRtcB2* **(B)** gene were normalized against those of RNA polymerase II and then are shown relative to psAP mock control. **(B, C)** Effects of *EhRtcB*s gene silencing on tRNA^Ile^(UAU) splicing. Unspliced and spliced tRNA^Ile^(UAU) were amplified by a combination of common forward and reverse primers **(B)** or by a combination of common forward and spliced form-specific reverse primers **(C)** and detected after electrophoresis. tRNA^His^(GUG) was used as negative control, which does not contain an intron (only in **B**). **(D)** Quantitation of spliced tRNA^Ile^(UAU) by qRT-PCR. The amounts of spliced tRNA^Ile^(UAU) from each gs strain were normalized against those of tRNA^His^(GUG) and then are shown relative to that in psAP mock control. **(E)** Growth kinetics of *EhRtcB*s gene silenced and control strains. The proliferation of trophozoites was monitored by cell number counting at 0, 24, 48, 72, and 96 h. Unpaired t-test was performed to determine all the statistically significant difference. P values are shown. ns, not significant.

In order to investigate if EhRtcBs are involved in tRNA splicing of tRNA^Ile^(UAU), transcriptional gene silencing *via* antisense small RNA was exploited. Since both *EhRtcB* genes are highly similar at the nucleic acid level (74.06% positional identity), we attempted to repress individual genes by producing anti-sense small RNA targeting either the entire protein-coding region or variable regions in the amino-terminal portion (first 264 and 298 nucleotides for *EhRtcB1* and *EhRtcB2*, respectively). Two N-terminal region targeting (psAP-RtcB1-N terminus and psAP-RtcB2-N terminus) and two full-length gene targeting (psAP-RtcB1-full length and psAP-RtcB2-full length) plasmids were introduced into parental G3 strain. Gene silencing was evaluated by qRT-PCR, and the transcript levels of *EhRtcB1* and *EhRtcB2* in the gene silenced strains are shown relative to those in the psAP mock control strain (**Figure 3A**). In the strains transformed with psAP-EhRtcB1-full length and psAP-RtcB1-N terminus, the transcript level of *EhRtcB1* was decreased by 92.5 ± 1.5% and 85.3 ± 5.6%, respectively, compared to psAP-mock control strain, while that of *EhRtcB2* remained unchanged. In contrast, when two plasmids designed to repress *EhRtcB2*, psAP-EhRtcB2-full length, and psAP-EhRtcB2-N terminus were introduced into G3, *EhRtcB2* transcript was almost completely abolished (98.9 ± 1.0% and 99.6 ± 0.2% decrease). However, repression of *EhRtcB1* gene expression (67 ± 25% decrease) was also observed in the strain transformed with psAP-RtcB2-full length, but not in the strain transfected with psAP-RtcB2-N terminus, due to the expected cross gene silencing.

We further investigated if *EhRtcB*s gene silencing affected tRNA processing (**Figures 3B, C**). In the psAP mock control strain, two bands were identified by polyacrylamide gel electrophoresis: a 62 bp fragment corresponding to the unprocessed tRNA^Ile^(UAU) and a 52 bp fragment corresponding to the spliced/ligated form (**Figure 3B**). In gene silenced strains, particularly the strains transfected with psAP-EhRtcB1-full length or psAP-EhRtcB1-short length, in which *EhRtcB1* gene expression was abolished or largely reduced, the amount of both the unprocessed and the spliced/ligated forms of tRNA^Ile^(UAU) transcript decreased (to the greatest extent for the spliced form in the strain transfected with psAP-EhRtcB1-full length). The decreased amount in unprocessed form is most likely due to the accelerated intron-splicing process when there is a lack of spliced/ligated forms of tRNA^Ile^(UAU). A slight decrease in the abundance of both the unprocessed and the spliced/ligated forms was also observed in the strain transfected with psAP-RtcB2-full length, in which *EhRtcB1* transcript decreased by about 75% (**Figure 3A**). Control tRNA, tRNA^His^(GUG), which is not subjected to splicing, was unchanged in all the strains. To further quantitate the amount of the spliced and functional tRNA^Ile^(UAU) transcript, the transcript was selectively PCR-amplified by common forward primer and spliced form-specific primer (**Figure 3C**). qRT-PCR showed that in the strains transfected with psAP-EhRtcB1gs-full length, psAP-EhRtcB1gs-N terminus, or psAP-EhRtcB2gs-N terminus, the amount of spliced tRNA^Ile^(UAU) was reduced by 94.0 ± 3.0%, 85.3 ± 5.6%, and 66.9 ± 25.3%, respectively (**Figure 3D**). In contrast, the amount of spliced tRNA^Ile^(UAU) was only reduced in the strain transfected with psAP-EhRtcB2gs-N terminus decreased by 16.1 ± 9.7%. The reduction of spliced tRNA^Ile^(UAU) agreed well with the reduction in the *EhRtcB1* gene transcript levels.

As for tRNA^Tyr^(GUA), similarly, two bands were amplified from cDNA in psAP mock control strain (**Figure S7**). A 65 bp upper band, which apparently has the same size as a band amplified from genomic DNA, corresponds to the unprocessed form, while a 52 bp band found in psAP-mock corresponds to the spliced form of tRNA^Tyr^(GUA). In psAP-EhRtcB1-full length, spliced tRNA^Tyr^(GUA) was abolished, and non-spliced tRNA^Tyr^(GUA) was largely diminished. Spliced form-specific amplification with various primer pairs to selectively target only the spliced form failed, which may be due to not-yet characterized base modifications or misprediction of the intron.

### EhRtcB1, but Not EhRtcB2, Is Essential for Amebic Proliferation

In order to confirm the essentiality of both EhRtcBs, we measured growth kinetics of the *EhRtcB1* and *EhRtcB2* gene silenced and control strains (**Figure 3E**). The strains that had been transfected with psAP-EhRtcB1gs-full length or psAP-EhRtcB1gs-N terminus, and showed *EhRtcB1*-specific gene repression, showed severe growth defects. In addition, the strain that had been transfected with psAP-EhRtcB2gs-full length and showed gene repression for both *EhRtcB1* and *EhRtcB2* also showed severe growth defects. These data indicate the essentiality of *EhRtcB1* and the unique non-overlapping role of EhRtcB1. In contrast, the strain that had been transfected with psAP-EhRtcB2gs-N terminus showed *EhRtcB2-*specific gene silencing, presented no growth retardation, suggesting non-essentiality of the gene under normal conditions.

### Cellular Localization of EhRtcB1 and EhRtcB2

**Figure 4:**
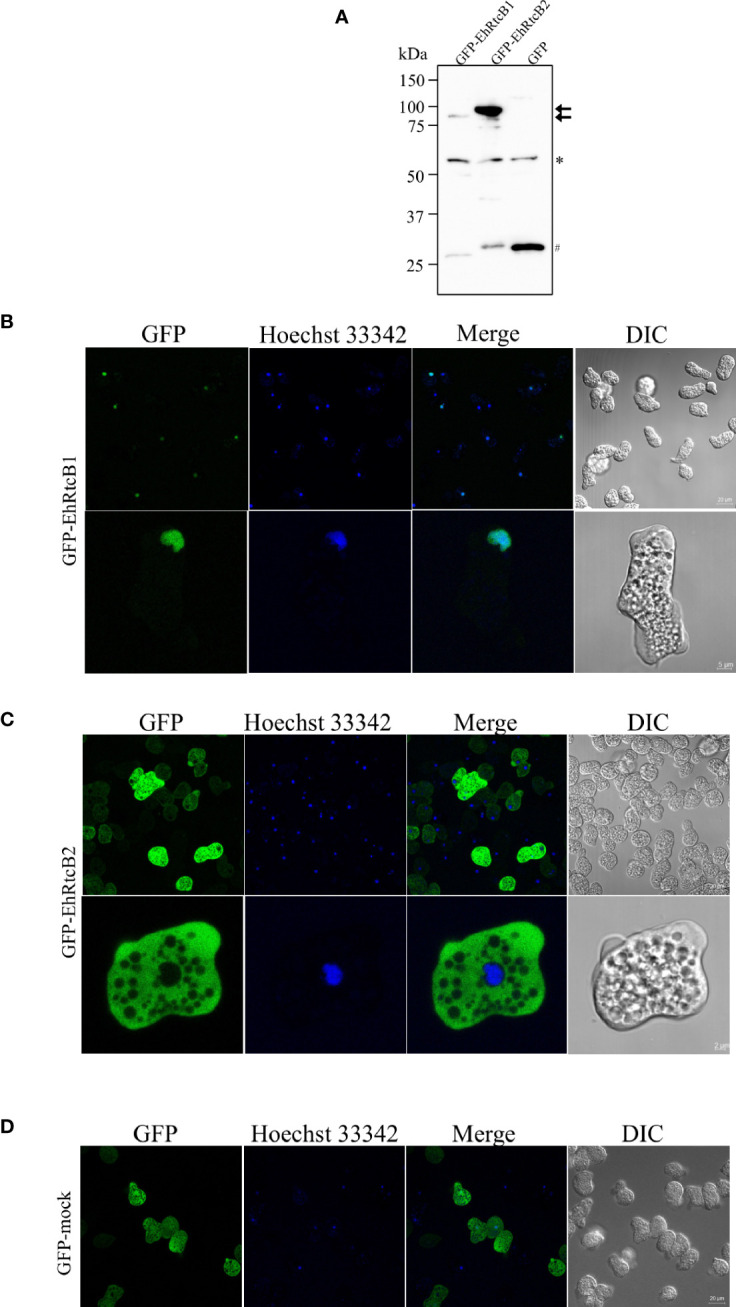
Expression and intracellular localization of GFP-fused EhRtcB1 and EhRtcB2. **(A)** Expression of GFP-RtcB1 and GFP-RtcB2 verified by SDS-PAGE and immunoblot analysis. Cell lysates from the transformant strains that expressed GFP-RtcB1 (86.5kDa) or GFP-RtcB2 (88.1kDa), cultivated with 20 μg/ml G418, were subjected to 10% SDS-PAGE and immunoblot analysis using anti-GFP antibody. Arrows indicate the untruncated GFP-EhRtcB1 and GFP-EhRtcB2. An asterisk indicates about 55 kDa non-specifically reacted unknown protein, while a sharp mark (#) indicates the fragments around 26 kDa corresponding to GFP, which were likely produced by cleavage of the fusion proteins. **(B–D)** Microscopic images of the amebic trophozoites expressing GFP-EhRtcB1 **(B)**, GFP-EhRtcB2 **(C)**, and GFP-Mock **(D)**. Images of live unfixed trophozoites stained with Hoechst (for nuclear DNA staining) captured by LSM 780 confocal microscope are shown with a low (upper panels) and high magnification (lower panels). GFP, Hoechst, merged, and differential interference contrast (DIC) images are shown.

To examine the distribution of EhRtcB1 and EhRtcB2 in the amebae, EhRtcB1 and EhRtcB2 fused with GFP at the amino terminus were ectopically expressed in *E. histolytica*. The expression of the proteins was confirmed by immunoblot analysis of amebic lysates, which reacted with the anti-GFP antibody (**Figure 4A**). GFP-EhRtcB1 and GFP-EhRtcB2 were recognized as the predominant bands of approximately 84 and 86 kDa, which agrees well with the predicted molecular weight of each protein (57.6 and 59.1 kDa, respectively) plus GFP (26.9 kDa). Also, note that truncated proteins, which likely correspond to only the GFP portion, was also detected in both transformant lines; however, in GFP-EhRtcB1 and GFP-EhRtcB2 expressing strain, the proportion of truncation was 32% and 7.6% of the combined amount of the full-length GFP-EhRtcB1 or GFP-EhRtcB2 and their truncation, based on the densitometric scanning of the blot. Immunofluorescence imaging revealed that GFP-EhRtcB1 was observed predominantly in the nucleus, while GFP-EhRtcB2 was evenly distributed in the cytoplasm (**Figures 4B, C**). In contrast, GFP was distributed in the whole cell in GFP-mock control strain (**Figure 4D**). Representative movies of time-lapse imaging of GFP-EhRtcB1 and GFP-EhRtcB2 are provided (**Movie S1**).

**Figure 5:**
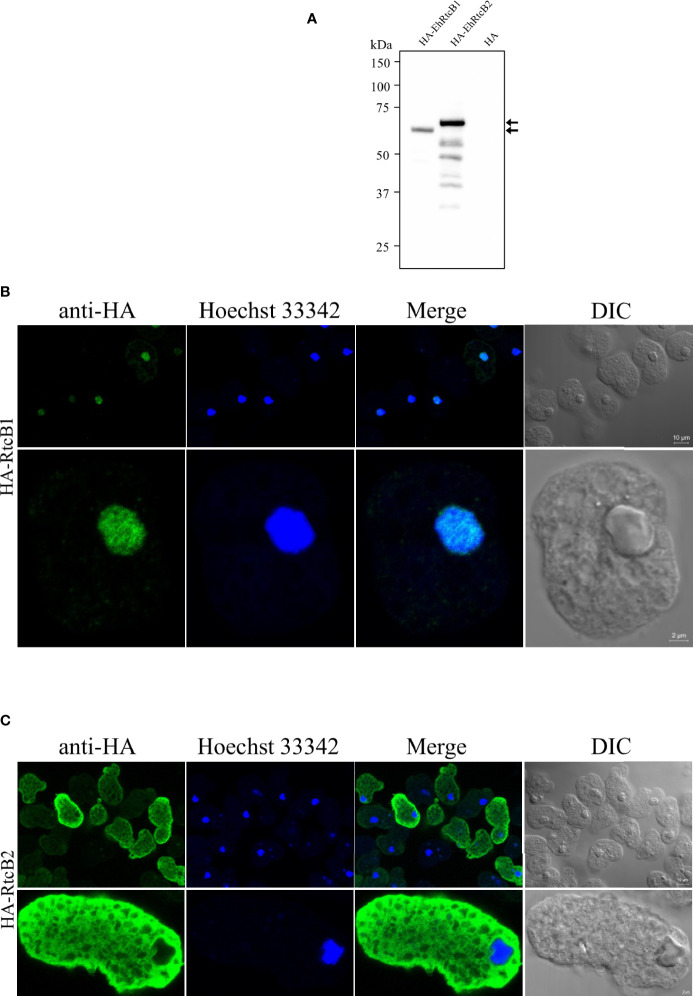
Expression and cellular localization of HA-tagged EhRtcB1 and EhRtcB2. **(A)** Expression of HA-RtcB1 and HA-RtcB2 verified by SDS-PAGE and immunoblot analysis. Cell lysates from the transformant strains that expressed HA-RtcB1 (61.3 kDa) or HA-RtcB2 (62.9 kDa) were subjected to SDS-PAGE and immunoblot analysis using anti-HA antibody. Arrows indicate the untruncated HA-EhRtcB1 and HA-EhRtcB2. **(B, C)** Microscopic images of the amebic trophozoites expressing HA-EhRtcB1 **(B)** and HA-EhRtcB 2 **(C)**. Images of trophozoites that were fixed, permeabilized, reacted with anti-HA primary antibodies, anti-mouse and anti-rabbit IgG secondary antibodies, and stained with Hoechst were captured on a LSM 780 confocal microscope. Images are shown with a low (upper panels) and high magnification (lower panels). HA, Hoechst, merged, and differential interference contrast (DIC) images are shown.

To exclude a possibility that the localization of GFP-fused RtcBs was influenced by GFP tagging or truncation of the fusion proteins, we also created amebae transformant strains that expressed HA-tagged EhRtcB1 and HA-EhRtcB2. The expression of HA-EhRtcB1 and HA-EhRtcB2 was validated by immunoblot analysis by anti-HA antibody (**Figure 5A**). HA-EhRtcB1 and HA-EhRtcB2 were recognized as the predominant bands of approximately 61 and 63 kDa, each corresponding to EhRtcB protein plus the HA tag (3.2 kDa). Also, note that several truncated proteins (around 28.8% of the total amount) were also detected in HA-EhRtcB2-expressing strain but not in HA-EhRtcB1-expressing strain. Immunofluorescence imaging of the fixed amebic transformants verified the disparate distribution of HA-EhRtcB1 and HA-EhRtcB2, indicated by live imaging described above (**Figures 5B, C**). The different localization of EhRtcB1 and EhRtcB2 indicates that these two proteins are likely involved in different biological functions in *E. histolytica*.

## Discussion

We have shown in this study that the *E. histolytica* genome encodes 2,670 tRNA genes, which include 42 tRNA^Tyr^(GUA) and 52 tRNA^Ile^(UAU) intron-containing genes, similar to the human genome (GTCh37/hg19) containing 5 tRNA^Ile^(UAU) and 13 tRNA^Tyr^(GUA) intron-containing genes (Chan and Lowe, 2009; Chan and Lowe, 2016). Consistently, we observed a dispersed organization of tRNA^Ile^(UAU) genes throughout the genome, while tRNA^Tyr^(GUA) genes were found in an array as previously reported (Clark et al., 2006; Tawari et al., 2008). This observation prompted us to search for tRNA processing enzymes in the *E. histolytica* genome. tRNA splicing plays a fundamental and essential role in life. RtcB/HSPC117 is known to be responsible for tRNA ligation after intron-cleavage in archeae and eukaryotes. However, RtcB/HSPC117 is not always conserved in these domains of life. In the protist *Trypanosoma brucei*, which belongs to Excavata, both RtcB and Trl1 are present, but only Trl1 mainly plays a role as tRNA ligase, while RtcB is suggested to be involved in RNA repair (Lopes et al., 2016). In this study, we have demonstrated that a representative protist that belongs to Amoebozoa, *E. histolytica*, possesses two distinct copies of RtcB but lacks Trl1. As far as we know, this is the first demonstration of the unicellular eukaryote that has two RtcBs that are differentially distributed in the cell but lacks Trl1.

Our results have shown that EhRtcB1 is involved in tRNA^Ile^(UAU) and tRNA^Tyr^(GUA) splicing by demonstrating that *EhRtcB1* gene silencing led to a reduction of the amount of spliced tRNA^Ile^(UAU) and tRNA^Tyr^(GUA). Consistently, gene silencing by psAP-EhRtcB2 full length, which cross-repressed EhRtcB1 expression, also led to a reduction of spliced tRNA^Ile^(UAU) and tRNA^Tyr^(GUA), but not tRNA^His^(GUG). Repression of *EhRtcB1* transcript also caused a reduction of unspliced form of tRNA^Ile^(UAU) and tRNA^Tyr^(GUA), but not tRNA^His^(GUG), which does not contain intron. This may be explained by the hypothesis that deprivation of mature tRNA^Ile^(UAU) in *EhRtcB1* transcript-reduced strains triggers an acceleration of the cleavage of precursor tRNA by endonuclease. Importantly, repression of EhRtcB1 transcript was associated with a reduction in the spliced form of tRNA^Ile^(UAU) and tRNA^Tyr^(GUA) and concomitant growth defect, reinforcing the essentiality of tRNA splicing in *E. histolytica.*

Although both EhRtcB1 (in cooperation with EhArchease) and EhRtcB2 possess RNA ligase activity *in vitro*, their distinct (nuclear and cytoplasmic) localization strongly suggests that they should be involved in different biological processes. The fact that gene-specific repression of *EhRtcB1*, but not *EhRtcB2*, caused proliferation defect reinforces the premise. While the biological role of EhRtcB1 was demonstrated to be the ligation of cleaved tRNA precursors in the nucleus, that of EhRtcB2 in the cytoplasm remains to be elucidated. Considering the facts that (1) EhRtcB2 catalyzes RNA ligation *in vitro* with or without EhArchease, (2) EhRtcB2 is not essential for proliferation under normal conditions, and (3) EhRtcB2 is localized in the cytoplasm, we propose that EhRtcB2 may play a role in mRNA ligation and RNA repair in the cytoplasm. Human HSPC117 is known to be localized both in the nucleus and cytoplasm and involved in, other than tRNA ligation, mRNA ligation. Two exons of *XBP1* gene transcript created by cleavage by IRE1α in a reaction involving unfolded protein response (UPR) are ligated by HSPC117 on the ER, and XBP1 finally participates in maintaining ER homeostasis. It was also demonstrated that human focal adhesion associated protein (FAAP), which is highly homologous to HSPC117 and located to the cytoplasm, is involved in cell adhesion by regulating the interactions and the dynamics of vinculin-paxillin and paxillin-focal adhesion kinase (FAK), which are responsible for recruitment of other adhesion-related proteins to the adhesion site and their dissociation from it (Hu et al., 2008). Alternatively, RtcB may be involved in the repair of the RNA that ends with 3’ phosphates when nucleotide acid damage happens, which canonical RNA ligases cannot repair. It was shown in *E. coli* that RtcB repairs a ribotoxin-induced breakage in a tRNA-like stem-loop structure (Tanaka and Shuman, 2011) and ribosomal RNA under antibiotic stress (Manwar et al., 2020). It was also demonstrated in yeast that RtcB carries out splicing of tRNA and HAC1 mRNA in a yeast strain lacking tRNA ligase Trl1 (Tanaka et al., 2011). Our data are also partially overlapped with the premise that RtcB is involved in a range of RNA processing involving tRNA, mRNA, and rRNA. In this context, the substrates of EhRtcB2 need to be experimentally demonstrated in the future. On the other hand, elucidation of conservation and evolution of HSPC117 across different domains of life is important to understand evolution of RNA maturation. It was previously shown that RNA polymerase III, which catalyzes tRNA transcription, is localized in the nucleus of *E. histolytica*, which was indirectly suggested by localization of its transcription factor, EhBRF (Jhingan et al., 2009). Taken together, these data suggest that both tRNA synthesis and splicing occur in the nucleus in *E. histolytica*.

Human RtcB, HSPC117, is known to form a complex with DDX1, CGI-99, FAM98B, ASW, and archease to exert the function of RNA ligation (Lu et al., 2014). Although archease is present in the genome and stimulates RNA ligase activity of EhRtcBs, as shown in this study, the other protein homologs (CGI-99, FAM98B, and ASW) except for DDX1 seem to be absent in *E. histolytica* genome. The potential complex formation of *E. histolytica* RtcB remains elusive. Identification of its cofactors can potentially lead to a better understanding of the evolution of tRNA splicing and diverse functions of RtcBs.

All in all, we have demonstrated that two RtcB proteins from *E. histolytica* possess RNA ligase activity. EhRtcB1 is located to the nucleus and involved in translation as tRNA ligase in cooperative action with co-factor, EhArchease, while EhRtcB2 is distributed to the cytoplasm and may be involved in mRNA splicing/ligation or nucleic acid repair as suggested in other organisms. Elucidation of the substrates of EhRtcB2, a repertoire of the components in EhRtcB1 complex, if present, and their functions requires further investigation.

## Data Availability Statement

The datasets presented in this study can be found in online repositories. The names of the repository/repositories and accession number(s) can be found in the article/**Supplementary Material**.

## Author Contributions

RP performed biochemical and cell biological experiments. GJ confirmed gene silenced strains. SY performed RNA self-ligation assay. RP, GJ, and SY analyzed data. TK-S conducted *in silico* analyses. RP and TN wrote the paper. All authors contributed to the article and approved the submitted version.

## Funding

This work was supported partly by Grant-in-Aid for Scientific Research (B) (JP18H0265, JP21H02723) to TN from the Japan Society for the Promotion of Science, and Grant for research on emerging and re-emerging infectious diseases from Japan Agency for Medical Research and Development (AMED, JP20fk0108138) to TN, Grant for Science and Technology Research Partnership for Sustainable Development (SATREPS) from AMED and Japan International Cooperation Agency (JICA) (JP20jm0110022) to TN.

## Conflict of Interest

The authors declare that the research was conducted in the absence of any commercial or financial relationships that could be construed as a potential conflict of interest.

## Publisher’s Note

All claims expressed in this article are solely those of the authors and do not necessarily represent those of their affiliated organizations, or those of the publisher, the editors and the reviewers. Any product that may be evaluated in this article, or claim that may be made by its manufacturer, is not guaranteed or endorsed by the publisher.

## Abbreviations

RtcB, RNA 2’,3’-cyclic phosphate and 5’-OH ligase; tRNA, transfer RNA; *Eh*, *Entamoeba histolytica*; tRNA^Ile^(UAU), isoleucine tRNA with UAU anticodon; tRNA^Tyr^(GUA), tyrosine tRNA with GUA anticodon; rRNA, ribosome RNA; *Hs*, *Homo sapiens*; *Ph*, *Pyrococcus horikoshii*; GPI, glycosylphosphatidylinositol; NLS, nuclear localization signal; qRT-PCR, real-time quantitative reverse transcriptase PCR; gs, gene silencing; GFP, green fluorescent protein; DDX1, DEAD-Box Helicase 1; XBP1, X-box binding protein 1; CGI-99, ORF names of RNA transcription, translation and transport factor protein; FAM98B, family with sequence similarity 98 member B; ASW, also known as chromosome 2 open reading frame 49; Trl1, tRNA ligase; PAGE, polyacrylamide gel electrophoresis.
