# Supplementary material for: Identification and Functional Characterization of Divergent 3’-Phosphate tRNA Ligase From Entamoeba histolytica
Source: Front Cell Infect Microbiol. 2021 Dec 17;11:746261. doi: 10.3389/fcimb.2021.746261 (PMC8718801; doi:10.3389/fcimb.2021.746261)
Supplement: Supplementary file 1 [file DataSheet_1.docx]

Supplementary Material

# Supplementary Figures and Tables

## Supplementary Figures

Alveolata

Viridiplantae

Excavata

Cryptophyta

Ochrophyta

Apusomonadida

Ochrophyta

Opisthokonta

Plantae

Amoebozoa

Rhizaria

Archaeplastida

Heterokonta

Alveolata

Microsporidia

*Entamoeba*

Heterokonta

Rhizaria

Archaeplastida

Archaea

Bacteria

Bacteria

**Supplementary Figure 1.** Phylogenetic reconstruction of RtcB from *Entamoeba* and other organisms.

BLASTp search was performed to identify HSPC117/RtcB orthologs from a broad range of taxa by using *Homo sapiens* HSPC117 as a query. Sequences were aligned by MAFFT and trimmed by TrimAl, and phylogenetic reconstruction was conducted by the maximum likelihood method in FastTree 2.1.10 with default parameters with HSPC117/RtcB orthologs got from BLASTp searches. Bootstrap proportions for the tree from 1000 replicates are shown in percentage at the nodes. A bar indicates 0.2 substitutions per site.

**Supplementary Figure 2.** Amino acid alignment of RtcBs.

EhRtcB1, EhRtcB2, *Pyrococcus horikoshii* RtcB, and *Homo sapiens* HSPC117 were aligned by clustalW. Red boxes indicate the conserved residues, D65-H404 dyad, implicated in guanylylation. Green boxes and dark blue boxes indicate the conserved Mn^2+^(I) and Mn^2+^(II) binding sites. A light blue box indicates the shared residue involved in two Mn^2+^ binding. A purple box indicates a potential NLS in EhRtcB1 predicted by NLS Mapper. Asterisks indicate the sites of identical residues. Colons and periods indicate the sites that show strong and mild conservation, respectively.

**Supplementary Figure 3.** Sequencing of tRNA^Ile^(UAU) transcripts.

PCR products targeting tRNA^Ile^(UAU) transcripts were amplified using cDNA from psAP-mock strain by common forward primer containing BamHI site and common reverse primer containing XhoI site. Amplified PCR products were cloned to a pET151/D-TOPO vector and sequenced as described in Materials and Methods. tRNAscan-SE predicted tRNA^Ile^(UAU) sequence from chromosome 1 was used as a reference. Primers and intron region are indicated.

**Supplementary Figure 4.** Visualization of unknown band in polyacrylamide gel but not in agarose gel.

(A) PCR products targeting tRNA^Ile^(UAU) were amplified using cDNA of psAP-Mock strain and loaded in a 3% agarose gel with different loading volume of 6 μl and 12 μl. ~120bp band is not visible in the agarose gel. (B) The same PCR products that were loaded in Figure S4(A) were loaded in a 10% polyacrylamide gel. ~120bp band appeared in polyacrylamide gel.

**5’- HO**

**OH -3’**

**+ pCp**

T4 RNA ligase I

**5’- HO**

**pCp -3’**

Circularization by RtcB

**pC**

**Supplementary Figure 5.** Strategy of RNA self-ligation assay.

A synthetic RNA with 5’- and 3’ hydroxyl termini was labelled with 5’-[^32^P]-pCp by using T4 RNA ligase 1. When the linear 5’-[32P]-pCp labelled RNA is ligated by RNA ligase, circular RNA is formed, which migrates faster than linear RNA in the urea PAGE.

**Supplementary Figure 6.** Alignment of tRNA^Ile^(UAU) genes and map of the oligonucleotide primers for tRNA^Ile^(UAU) amplification.

Sequence alignment of all 7 representative pre-tRNA^Ile^(UAU) genes from the *E. histolytica* genome. Forward and reverse primers used in RT-PCR are shown.

psAP-Mock

EhRtcB1gs-full length

EhRtcB1gs-N-terminus

EhRtcB2gs-full length

EhRtcB2gs-N-terminus

Genomic DNA

tRNA^Tyr^(GUA) Common forward primer + Common reverse primer

Marker

Marker

cDNA

RNA without RT

psAP-Mock

EhRtcB1gs-full length

EhRtcB1gs-N-terminus fragment

EhRtcB2gs-full length

EhRtcB2gs-N-terminus fragment

60bp

80bp

40bp

Spliced tRNA^Tyr^(GUA)

Unspliced tRNA^Tyr^(GUA)


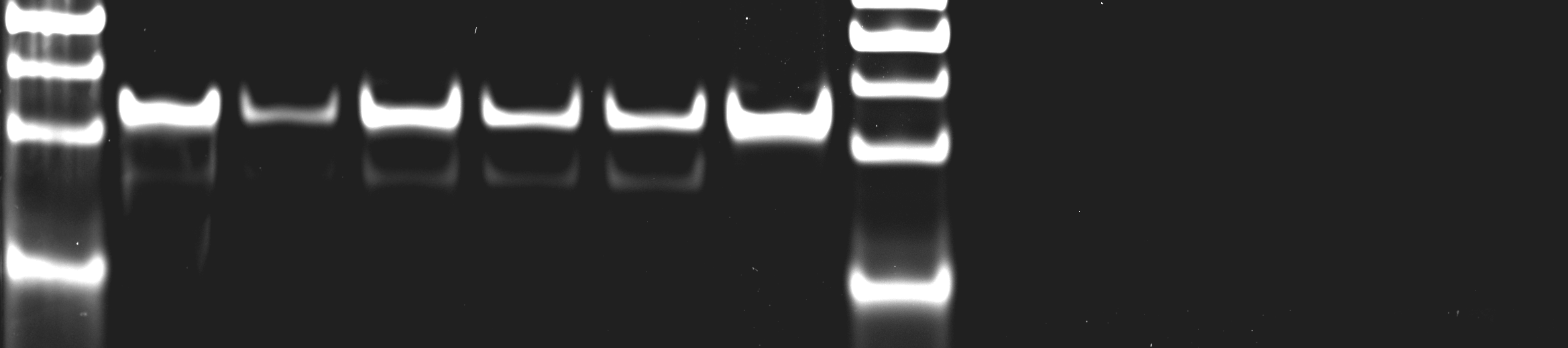


**Supplementary Figure 7.** Effects of *EhRtcB*s gene silencing on tRNA^Tyr^ (GUA) splicing. Unspliced and spliced tRNA^Ile^ (UAU) were amplified by a combination of common forward and reverse primers and detected after electrophoresis. Genomic DNA was also used to amplify the intron-containing region of the gene. Note that the PCR reactions using RNA samples without reverse transcriptase were also electrophoresed as negative control.

## Supplementary Table

**Supplementary Table 1.** Primers used in the study

# Supplementary Movie

**Supplementary Movie 1.** Movies showing time-lapse live imaging showing cellular localizations of GFP-EhRtcB1 (A, B) and GFP-EhRtcB2 (C, D) under the LSM 780 confocal with low (A, C) and high magnification (B, D).
